# Negative appendicectomy rate as a quality metric in the management of appendicitis: impact of computed tomography, Alvarado score and the definition of negative appendicectomy

**DOI:** 10.1308/003588412X13171221592131

**Published:** 2012-09

**Authors:** JG Mariadason, WN Wang, MK Wallack, A Belmonte, H Matari

**Affiliations:** Metropolitan Hospital, New York,US

**Keywords:** Appendicitis, Radiography, Differential diagnosis, Decision support techniques, Computed tomography

## Abstract

**INTRODUCTION:**

The negative appendicectomy rate (NAR) is a quality metric in the management of appendicitis. While computed tomography (CT) has been associated with a low NAR, Alvarado scoring produces an acceptable NAR. The definition of negative appendicectomy may affect the NAR. This study examined the impact of CT, Alvarado score and definition on the NAR.

**METHODS:**

The charts of 1,306 emergency appendicectomy patients from 1996 to 2010 were reviewed. Three five-year cohorts were created (Cohort A: 1996–2000, Cohort B: 2001–2005, Cohort C: 2006–2010) and the NAR was calculated for each cohort using two definitions of negative appendicectomy: absence of inflammation (NAR-STD) and absence of intramural neutrophils (NAR-STR). NAR-STD was correlated to the CT rate for Cohorts B and C and also to Alvarado score for Cohort C.

**RESULTS:**

When the definition of negative appendicectomy was changed, the NAR rose from 9.2% to 15.8% (*p*=0.0097) for Cohort A, from 2.8% to 8.6% (*p*=0.0180) for Cohort B (CT rate: 80.6%) and from 3.0% to 6.7% (*p*=0.0255) for Cohort C (CT rate: 92.4%). The introduction of CT lowered NAR-STD from 1996–2000 (9.2%) to 2001–2010 (2.9%) but increasing the CT rate from 2001–2010 had no impact on the NAR. The positive predictive value for Alvarado score (98.60%) and CT (99.03%) were similar.

**CONCLUSIONS:**

The definition of a negative appendicectomy determines the NAR. CT reduces the NAR regardless of definition but routine CT is unnecessary for male patients with positive Alvarado scores. Early/mild appendicitis may resolve without surgery and CT may contribute to unnecessary surgery. Alvarado scoring allows selective use of CT in suspected appendicitis.

The annual appendicectomy volume cited for the US in 1997 was 250,000 cases with a negative appendicectomy rate (NAR) of 15%.^1,2^ In 2007 there were 326,000 appendicectomies.^3^ Several recent papers have cited a declining NAR, including several large database studies and meta-analyses with NARs as low as 6–8%^4–8^ and single institution studies with NARs as low as 1.7–7%,^9–13^ coinciding with the increased use of computed tomography (CT) and laparoscopy. While CT is often credited with lowering the NAR,^4,5,8–15^ a definitive causal relationship has not been established and lingering questions about proper usage remain.^14–17^

CT has become routine in the management of suspected appendicitis at many US institutions and the current debate is whether imaging should even be mandatory.^12,13^ Several centres continue to report NARs as high as 17–36% without the use of CT.^18–22^ Some series have championed the virtues of clinical scoring systems like those of Teicher, Alvarado, Lintula and Tzanakis^23–27^ in reducing NARs. The Alvarado scoring system in particular has yielded NARs as low as 6–8%.^25,26^ A clinical pathway where CT was used selectively produced a reduction of the NAR from 16% to 4%.^15^

As we were aware that our NAR for the past ten years was quite low, we decided to study the effect of CT, clinical findings and laparoscopy on our NAR. In the literature, different criteria were used to define appendicitis and negative appendicectomy with the most common definition being: ‘Negative appendicectomy is the absence of inflammation or pathology in the appendix.’ We compared our NAR using this definition of negative appendicectomy (NAR-STD) to our NAR with a more stringent definition (NAR-STR) employed by a few authors,^10,34^ namely: ‘Negative appendicectomy is the absence of intramural neutrophils in the appendix.’

## Methods

The study was performed at Metropolitan Hospital, a municipal hospital in New York that serves a largely Hispanic immigrant population with a full-time faculty of general surgeons supported by surgical residents from New York Medical College. The pathology records (computerised since 1996) were searched for all appendicectomies carried out at Metropolitan Hospital during the 15-year period from 1996 to 2010. Incidental appendicectomies were excluded. A chart review was conducted on the remaining cases and interval appendicectomies were then also excluded, leaving 1,306 patients operated on emergently for a diagnosis of acute appendicitis.

The years 1996 to 2000 only had data on sex, age and indication for surgery to be correlated with pathology. This constituted Cohort A and represented the pre-CT era although CT was performed in a few cases. Cohort B (2001–2005) provided complete laboratory and radiological data but incomplete clinical data so only sex, age and CT findings could be correlated with pathology. Cohort C (2006–2010) yielded detailed clinical, laboratory, radiological and operative surgery data and was studied for sex, age, Alvarado score, CT findings and type of surgery (open versus laparoscopic) in relation to pathology.

NARs were compared using standard criteria (NAR-STD) versus more stringent criteria (NAR-STR) for the entire 15-year period. NAR-STD was correlated to CT For Cohorts B and C, and to CT and Alvarado score for Cohort C. Type of surgery was also examined for Cohort C.

### Definitions

Appendicitis was defined for the main study as the presence of inflammatory cells (polymorphonuclear leucocytes, lymphocytes or plasma cells) in the appendix. The absence of inflammation was considered a negative appendicectomy (NAR-STD). In the more stringent definition, the absence of polymorphonuclear leucocytes in the wall of the appendix was considered a negative appendicectomy (NAR-STR).

The Alvarado score was calculated retrospectively as per Alvarado’s description^24^ with one modification: right lower quadrant pain was sometimes substituted for migratory pain due to imprecise documentation of pain (Table 1).

**Table 1:** Modified Alvarado score

Signs/symptoms	Score
Migratory or right lower quadrant pain	1
Anorexia/acetonuria	1
Nausea/vomiting	1
Tenderness in right lower quadrant	2
Rebound tenderness	1
Elevated temperature	1
Leucocytosis	2
Shift	1
	
Negative	1–4
Equivocal	5–6
Positive	7–10

CT was considered positive if read as consistent with, compatible with or demonstrating acute appendicitis and criteria included periappendiceal stranding, an enlarged and swollen appendix with or without a faecalith. Phrases like ‘cannot exclude appendicitis’, ‘correlate clinically’ or ‘suggest follow-up study’ were considered equivocal and readings of ‘negative for appendicitis’ or ‘normal appendix’ were taken as negative.

### Statistical analysis

NAR-STD and NAR-STR were compared using an unpaired t-test with two-tailed *p*-value calculation and a one-way analysis of variance. Pearson’s correlation coefficients were calculated to analyse the relationship between CT rate and NAR-STD for Cohort B.

## Results

A total of 1,306 charts were reviewed and 3 cohorts formed as follows:

Cohort A (1996–2000, pre-CT) consisted of 380 patients: 231 male and 149 female. Changing the definition from NAR-STD to NAR-STR increased the overall NAR from 9.2% to 15.8%, a significant change (*p*=0.0097). For male patients it increased the NAR from 6.1% to 10.4% (*p*=0.1976) and for female patients from 14.1% to 24.2% (*p*=0.1053), neither of these changes being significant (Table 2).

**Table 2:** Comparison of results depending on different definitions for negative appendicectomy

Year	NAR-STD		NAR-STR	
	Male	Female	Male	Female
1996	1	7	1	14
1997	5	4	8	10
1998	2	3	3	4
1999	4	4	6	4
2000	2	3	6	5
**1996–2000**	**14/231 (6.1%)**	**21/149 (14.1%)**	**24/231 (10.4%)**	**36/149 (24.2%)**
	**Overall: 35/380 (9.2%)**		**Overall: 60/380 (15.8%)**	
				
2001	2	2	6	4
2002	0	1	6	4
2003	1	1	1	3
2004	3	2	4	5
2005	1	0	5	2
**2001–2005**	**7/308 (2.3%)**	**6/156 (3.8%)**	**22/308 (7.1%)**	**18/156 (11.5%)**
	**Overall: 13/464 (2.8%)**		**Overall: 40/464 (8.6%)**	
				
2006	2	0	3	2
2007	0	1	4	3
2008	1	4	5	5
2009	2	1	3	2
2010	0	3	1	3
**2006–2010**	**5/297 (1.7%)**	**9/165 (5.5%)**	**16/297 (5.4%)**	**15/165 (9.1%)**
	**Overall: 14/462 (3.0%)**		**Overall: 31/462 (6.7%)**	

NAR-STD = standard definition of negative appendectomy rate; NAR-STR = tringent definition of negative appendectomy rate

Cohort B (2001–2005, CT rate: 80.6%) consisted of 464 patients: 308 male and 156 female. Changing the definition from NAR-STD to NAR-STR increased the overall NAR from 2.8% to 8.6% (*p*=0.0180). For male patients it increased the NAR from 2.3% to 7.1% (*p*=0.0256) and for female patients from 3.8% to 11.5% (*p*=0.0011), both changes being statistically significant.

Cohort C (2006–2010, CT rate: 92.4%) consisted of 462 patients: 297 male and 165 female. Changing the definition from NAR-STD to NAR-STR increased the overall NAR from 3.0% to 6.7% (*p*=0.0255). For male patients it increased the NAR from 1.7% to 5.4% (*p*=0.0247) and for female patients from 5.5% to 9.1% (*p*=0.273). The change was significant for males but not for females, who had five true negative CT findings overruled, perhaps explaining the aberration. The false positive CT rate was 1.3% (positive predictive value [PPV]: 99.03% for male and 97.20% for female patients, sensitivity: 99.03% for males and 93.65% for females). The false positive rate for the Alvarado score was 1.9% (PPV: 98.96% for male and 96.69% for female patients, sensitivity: 92.23% for males and 92.26% for females). Thirteen patients in Cohort C had false negative CT findings and would not have been operated on, with potentially serious consequences. Similarly, 23 patients had false negative Alvarado scores (Table 3).

**Table 3:** Comparison of computed tomography and Alvarado score

Year	CT false negative	CT false positive	Alvarado false negative	Alvarado false positive
2006	1	0	5/6	1/70
2007	3	1	9/11	0/63
2008	5	0	3/3	4/69
2009	2	2	2/3	1/85
2010	2	2	4/6	1/74
				
Total	13	5/381 (1.3%)	23	7/361 (1.9%)
	Total negative: 17	Total positive: 381	Total negative: 29	Total positive: 361
	True negative: 4	True positive: 376	True negative: 6	True positive: 354
		PPV: 98.20%		PPV: 98.00%
				
		Equivocal: 29	Equivocal: 72	
			Negative appendicectomies: 10	
	CT erroneous (2006–2010): 18/428 (4.2%)			
	Alvarado erroneous: 30/462 (6.5%)			

CT = computed tomography; PPV = positive predictive value

**Figure 1:**
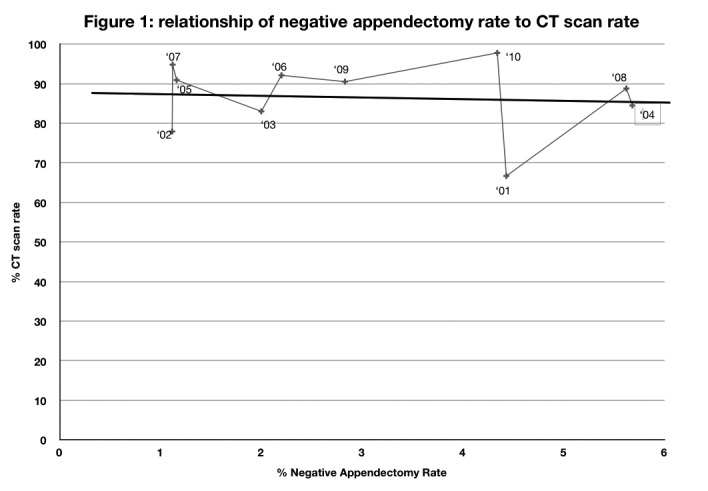
Computed tomography rate compared with negative appendicectomy rate. Numbers on graph represent the years 2001–2010.

The impact of CT is evident in the change in NAR from Cohort A to B but an increasing CT rate made no difference to the NAR in the period 2001–2010 (*p*=0.0120) although annual NARs of 1.2%, 2.0%, 1.1%, 2.2% and 1.1% (ie comparable to the best reported NAR) were recorded during this period (Fig 1 and Table 4).

**Table 4:** Breakdown of computed tomography (CT) use and negative appendicectomy rate (standard definition)

Year	Total number of patients	CT performed	No CT	Negative appendicectomy
1996	74			8 (10.8%)
1997	81			9 (11.1%)
1998	57			5 (8.8%)
1999	74			8 (10.8%)
2000	94			5 (5.3%)
**1996–2000**	**380**			**35 (9.2%)**
Breakdown	231 male (61%)			
	149 female (39%)			
				
2001	93	62	31	4 (4.3%)
2002	86	67	21	1 (1.2%)
2003	100	83	17	2 (2.0%)
2004	97	82	16	5 (5.2%)
2005	88	80	8	1 (1.1%)
2006	89	82	7	2 (2.2%)
2007	88	83	5	1 (1.1%)
2008	89	79	10	5 (5.6%)
2009	106	96	10	3 (2.8%)
2010	90	88	2	3 (3.3%)
**2001–2010**	**926**	**801 (86.5%)**	**125 (13.5%)**	**27 (2.9%)**
Breakdown	605 male (65%)	499 male	106 male	
	321 female (35%)	302 female	19 female	
				
**2001–2005**	**464**	**374 (80.6%)**	**90 (19.4%)**	**13 (2.8%)**
Breakdown	308 male (66%)	232 male	76 male	
	156 female (34%)	142 female	14 female	
**2006–2010**	**462**	**427 (92.4%)**	**35 (7.6%)**	**14 (3.0%)**
Breakdown	297 male (64%)	267 male	30 male	
	165 female (36%)	160 female	5 female	

For Cohorts B and C, changing the definition of negative appendicectomy significantly altered the NAR overall. For male patients, there was significant improvement in NAR-STD from A to C (*p*<0.05) but not from A to B or B to C, suggesting that improving CT had an impact in recent years. For female patients, there was significant improvement in NAR-STD from A to B (*p*<0.05) and A to C (*p*<0.05) but not B to C, probably because 5/9 negative appendicectomies among females in Cohort C were in patients with true negative CT findings that were overruled.

Perforation rates for Cohorts A, B and C were statistically unchanged (*p*=0.65) at 14.5%, 12.8% and 13.8% respectively. Our laparoscopic appendicectomy rate increased from 23% in 2006 to 75% in 2010, without significant effect on negative appendicectomy or perforation rates. A reduction in wound infections, length of hospital stay and return-to-work time was observed but not studied.

In Cohort C, the patients were also studied by sex and age to see if there was a difference in accuracy of CT for male and female patients under and over the age of 14 years. This was considered an average age for puberty and sexual activity, which introduce into the differential diagnosis conditions that mimic appendicitis. Overall CT accuracy was 95.0%; it was 97.2% for male and 90.4% for female patients, regardless of age.

With the more stringent definition of negative appendicectomy used by some authors,^10,38^ our NAR rose from 9.2% to 15.7% for Cohort A, from 2.8% to 8.6% for Cohort B and from 3.0% to 6.7% for Cohort C, all significant changes. The overall NAR for 2001–2010 rose from 2.9% to 7.9%.

## Discussion

Approximately 250,000 cases of appendicitis occurred annually between 1979 and 1984 in the US.^1^ During that period, the incidence of appendicitis decreased by 14.6%. Flum and Koepsell reported that in 1997 a total of 261,134 patients had non-incidental appendicectomies with a 15.3% NAR.^2^ In 2007, 326,000 appendicectomies were performed in the US, making appendicectomy still the most common general surgical emergency operation.^3^

At Metropolitan Hospital the number of appendicectomies rose from 380 in the first 5 years of the study to 464 in the middle 5 years, remaining at 462 in the last 5 years. Meanwhile, the NAR as commonly defined (NAR-STD) decreased. While CT use was beneficial in lowering the NAR from the 9.2% of the pre-CT era (1996–2000) to 3.0% in the subsequent periods (2001–2010) (*p*=0.0120), the increasing CT rate from 66% to 98% did not further reduce NAR-STD and no correlation between increased CT rate and lower NAR was demonstrable (Pearson correlation coefficient r ꞊-0.1049).

### Computed tomography

Several large database studies, meta-analyses and single institution studies credit CT with reducing the NAR,^4,5,8–15^ especially for women in the reproductive years. Some even suggest that CT should be made routine in the evaluation of suspected appendicitis.^12,13^ Since the landmark study of Rao *et al*,^9^ CT rates in the US have risen exponentially and NARs of 1–3% have been reported.^10,11^

CT has great value in ruling out appendicitis. It can also identify conditions suitable for conservative management such as gynaecological diseases, intra-abdominal fat necrosis and appendiceal abscess. Indeed, with interval appendicectomy no longer considered mandatory,^31,32^ surgery may be avoided altogether. The main disadvantages of CT are cost, delay in time to surgery and radiation exposure, especially for children. Costs are generally offset by shorter hospital stay and fewer negative appendicectomies. Delay is no longer an issue in most institutions. Radiation remains a concern and efforts to reduce this are under study.

Our own experience has been that CT was important in reducing the NAR by any definition. Low NAR (4.3%) was achieved in 2001 when only 67% of patients received CT and a significant number of readings were overruled by surgeons. Multidetector CT (16-slice, radiation dose 23.68mGy) became available to us in 2004 with a sharp decline in the number of false negative findings. Increasing the CT rate to almost 100% did not improve the NAR. Our study of NAR-STD versus NAR-STR suggests that CT may be overly sensitive, detecting potentially self-limiting cases of mild or resolving appendicitis. Indeed, conservative management of appendicitis has been reported with some success.^33–35^ Positive CT findings may therefore prompt unnecessary surgery.

### Alvarado score

Most surgeons pride themselves on their ability to diagnose appendicitis without resorting to scoring systems but NARs using ‘clinical judgement’ have been as high as 17–36%.^18–22^ With its inherent discipline, the Alvarado score has produced acceptable NARs of <8%,^25,26^ making it a valuable tool either for screening or as an alternative to CT or ultrasonography.

Petrosyan *et al* incorporated Alvarado scores and CT into the management of 1,630 patients with right lower quadrant pain and suspected appendicitis.^16^ CT was performed in 56%, sparingly for Alvarado scores of 8–10, somewhat more frequently for scores of ≤4 and commonly for scores of 5–7. The overall NAR was 6%, regardless of whether CT was performed or not. The biggest impact of CT was on the Alvarado 5–7 group, where the addition of CT reduced the NAR from 6.2% to 3.3%. In the prospective study of Antevil *et al*, a reduction in NAR from 16% to 4% was achieved after implementing a pathway that included early surgical evaluation and CT for all female patients and only male patients with low suspicion for acute appendicitis.^17^

### Ultrasonography

Ultrasonography was not studied in this paper. It has not been shown to be as accurate as CT but poses no radiation risk and is particularly attractive for children, for whom a pathway incorporating clinical examination with ultrasonography has had some success.^38–40^

### Alvarado score vs computed tomography

Our study demonstrated that in 361 of the 462 patients with positive Alvarado scores (76.1%), a NAR of 1.9% can be maintained. During the same period, in 381 of the 462 patients with positive CT findings (82.4%), the NAR was 1.3%. The difference was slight and CT was not necessary in many patients but reinforced the surgeon’s decision to operate. Male patients with positive Alvarado scores (PPV: 99.96%) gained little from CT (PPV: 99.03%). For females, positive Alvarado scores (PPV: 96.69%) and positive CT findings (PPV: 97.52%) were surprisingly close, suggesting that the Alvarado score is valuable for females too.

Overall, however, both Alvarado scores and CT were less reliable for females than for males and the combination of clinical findings and CT was important in reducing the NAR in female patients. A positive Alvarado score was a good predictor of appendicitis, especially for males in this study. Although CT added to accuracy, overruling falsely negative CT findings was an important part of our low NAR. Equivocal and negative Alvarado scores were less accurate and CT was important in identifying many cases of appendicitis.

We believe that CT should be performed in females during the reproductive years, in males with equivocal Alvarado scores and in patients with symptoms for over 48 hours or clinical indications of abscess formation.

### Laparoscopy

Laparoscopy did not reduce the NAR in our study or in most others but it did improve outcomes, shortening hospital stay and reducing wound infections.^29–32^ Laparoscopy has become the preferred surgical approach at many institutions including ours and a negative laparoscopy without completing an appendicectomy could lower the NAR^5^ but it would still be an unnecessary procedure.

### Negative appendicectomy rate

The preponderance of male patients no doubt contributed to our low NAR. Nevertheless, we demonstrated that in an urban medical centre, where surgical residents make the preliminary decision to operate for appendicitis, it is possible to maintain a NAR of less than 3% without routine use of CT. Male patients with high Alvarado scores do not require CT.

The NAR depends on the definitions of ‘acute appendicitis’ and ‘negative appendicectomy’. The more liberal definitions of NAR used by many authors^4,6,9,11,13,14,16^ would logically produce lower NARs and our study confirms this. Many reports, especially meta-analyses and database studies, do not define appendicitis or negative appendicectomy clearly.^5,8,12,15,17^ When a more stringent definition of negative appendicectomy adopted by a few authors^10,38^ was used, our overall NAR rose significantly. Without diminishing the low NAR achieved by some institutions, it may explain discrepancies in NAR between reported series.

It also raises the question of whether the NAR is the appropriate quality metric and whether CT lowers the NAR while increasing the rate of unnecessary surgery. Our finding of significant change in the NAR depending on definition (NAR-STD vs NAR-STR) in the CT era implies this is so. For males in this study, there was no significant change in NAR from Cohorts A to B or B to C, suggesting a subtle impact of CT. Combined with the finding of significant change in the NAR depending on definition for Cohorts B and C, it indicates that CT may be too sensitive, identifying cases of self-limiting appendicitis that are suitable for non-operative treatment. Primary non-operative management of appendicitis has been reported in some series with a modicum of success.^31–33^

### Limitations

This study suffers from the same limitations as studies like the Surgical Care and Outcomes Assessment Program trial^4^ in that appendicectomy specimens were used to capture the cases. Patients with suspected appendicitis but ruled out by negative CT findings were therefore not captured and the negative predictive value, the specificity and the full benefit of liberal CT scanning could not be measured.

## Conclusions

The NAR is a flawed quality metric that depends on the definition of ‘acute appendicitis’ and ‘negative appendicectomy’. CT reduces the NAR but routine CT is unnecessary to maintain a NAR below 3% and for male patients a positive Alvarado score suffices. The Alvarado score is a valuable tool in diagnosing appendicitis. Overuse of CT may contribute to unnecessary surgery.

**Figure 2:**
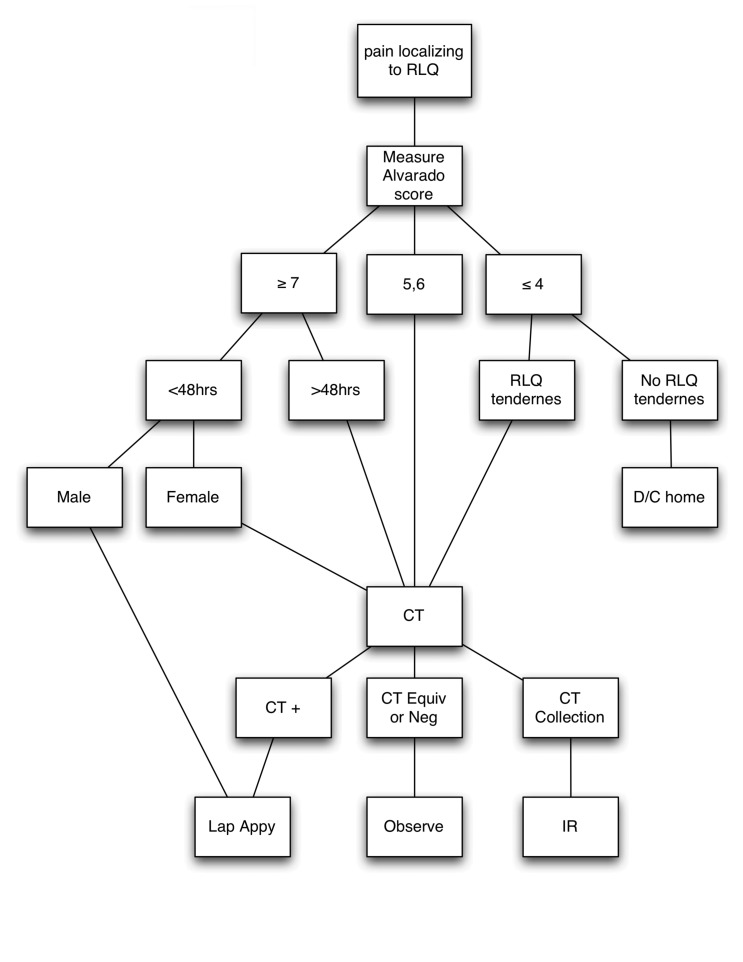
Algorithm for management of suspected appendicitis

An algorithm combining Alvarado score with selective use of CT is suggested (Fig 2).

RLQ = right lower quadrant; CT = computed tomography; pos = positive; equiv = equivocal; neg = negative; Lap app = laparoscopic appendicectomy; IR = interventional radiology
